# Diabetes Status Modifies the Association Between Carotid Plaque Burden and Retinal Microvascular Parameters

**DOI:** 10.7150/ijms.130441

**Published:** 2026-04-08

**Authors:** Ming Yang, Xueru Cheng, Jiaxin Guan, Zixuan Zhou, Jialin Wang

**Affiliations:** Department of Ophthalmology, Beijing Friendship Hospital, Capital Medical University, NO.95 Yong'an Road, Xicheng District, Beijing 100050, China.

**Keywords:** carotid plaque burden, diabetes mellitus, retinal microvascular parameters

## Abstract

**Purpose:**

Carotid plaque burden (CPB) is a key indicator of atherosclerosis, yet its relationship with ocular microcirculation in the context of type 2 diabetes mellitus (T2DM) remains poorly understood. This study aimed to examine the association between CPB and ocular vascular parameters, and to evaluate whether this relationship is modified by diabetic status.

**Methods:**

This retrospective study included 237 eyes from 123 participants (47 with T2DM, 76 without diabetes). All participants underwent head and neck computed tomography angiography (CTA) to quantify CPB using plaque presence and maximum thickness scores. Retinal vascular calibers (central retinal arteriolar equivalent [CRAE], central retinal venular equivalent [CRVE], and arteriovenous ratio [AVR]) were assessed using fundus photography. Hemodynamic parameters (peak systolic velocity [PSV], end-diastolic velocity [EDV], resistance index [RI], and pulsatility index [PI]) of the ophthalmic artery (OA), central retinal artery (CRA), and posterior ciliary artery (PCA) were measured using color Doppler ultrasound. Linear mixed-effects models (LMM) with patient-level random intercepts were employed to account for inter-eye correlation, with adjustment for age, sex, hypertension, smoking history, ischemic heart disease, stroke history, and hyperlipidemia.

**Results:**

In the diabetic group, plaque thickness score was significantly associated with CRAE (beta = -2.223, P = 0.003), AVR (beta = -0.011, P < 0.001), and PCA RI (beta = 0.005, P = 0.021). Plaque presence score was significantly associated with AVR (beta = -0.022, P = 0.021) and PCA RI (beta = 0.016, P = 0.009). No such associations were observed in the non-diabetic group (all P > 0.05). Interaction analyses revealed that diabetic status significantly modified the relationship between plaque thickness and both CRAE (P for interaction = 0.014) and AVR (P for interaction = 0.002). For plaque presence score, diabetic status significantly modified the association with AVR (P for interaction = 0.013).

**Conclusion:**

Diabetic status significantly modifies the associations between CPB and retinal microvascular parameters (CRAE and AVR). These findings underscore the importance of metabolic status in ocular vascular pathology and suggest that diabetic patients may benefit from intensified ocular monitoring even with modest CPB. Further longitudinal studies are warranted to elucidate the underlying mechanisms.

## Introduction

Diabetes mellitus (DM) is a metabolic disorder characterized by chronic hyperglycemia. Its global prevalence continues to rise, with an estimated 537 million cases in 2021, projected to increase to 643 million by 2030 and 783 million by 2045 1. DM is a significant risk factor for systemic vascular disease, contributing to microvascular complications (diabetic nephropathy, retinopathy, and neuropathy) and macrovascular complications (stroke and coronary artery disease), which may lead to disability and death, imposing a substantial economic burden on individuals, families, and healthcare systems 2-3. Carotid plaques can induce carotid artery stenosis, resulting in diminished ocular blood perfusion and alterations in hemodynamics. It is well-established that type 2 diabetes mellitus (T2DM) accelerates the progression of atherosclerosis. Even short-term diabetes may contribute to an elevated CPB, adverse carotid remodeling, and luminal narrowing. A study by Jocelyn *et al*. reported that carotid artery stenosis and wall thickening in patients with T2DM were associated with a reduction in perifoveal microvascular density 4. Robyn *et al*. observed that diabetic patients displayed greater retinal venous tortuosity compared to non-diabetic subjects; however, the positive correlation between tortuosity and glycated hemoglobin (HbA1c) was less pronounced in the diabetic group, indicating potential impairment of retinal vascular autoregulation in diabetes 5.

CPB serves as a critical metric for evaluating the total extent of carotid atherosclerosis and is significant in predicting and mitigating major adverse cardiovascular events 6. The OA, being the first branch of the internal carotid artery, derives its perfusion directly from upstream hemodynamic conditions. As the principal source of ocular blood supply, the OA is capable of reflecting changes in ocular blood flow in a more immediate and dynamic manner 7. The retinal artery, which terminates from the OA, along with the short posterior ciliary arteries—responsible primarily for supplying the choroidal circulation—collectively form the terminal vascular system of the carotid artery and exhibit high sensitivity to hemodynamic fluctuations 8.

Current understanding of the relationship between CPB and ocular vascular parameters remains limited, particularly concerning the moderating influence of diabetic status, which has yet to be fully elucidated. This study aims to examine the association between CPB and both morphological and hemodynamic parameters of the ocular vasculature, and to evaluate whether this relationship is modulated by diabetic status.

## Methods

### Study population

This retrospective study was approved by the Ethics Committee of Beijing Friendship Hospital, Capital Medical University (No. 2020-P2-008). A total of 237 eyes from 123 patients were included.

Inclusion Criteria: Patients who underwent head and neck CT angiography (CTA) and ophthalmic examinations (fundus photography and ocular ultrasound flowmetry) at Beijing Friendship Hospital between January 2014 and December 2019 due to suspected carotid artery disease were enrolled. Based on WHO criteria for type 2 diabetes, patients were stratified into diabetes (DM) and non-diabetes (non-DM) groups. Exclusion Criteria: Patients with diabetic retinopathy (DR) or diabetes duration exceeding 10 years; Ocular pathologies affecting measurements (e.g., severe cataracts, corneal lesions, high myopia, glaucoma, macular degeneration, retinal vascular occlusion, optic nerve disorders); Ophthalmic artery blood flow reflux; Recent (within 3 months) acute cardiovascular or cerebrovascular events (e.g., myocardial infarction, stroke); Systemic infections or malignancies. All participants provided informed consent and were aware of the study's nature and implications.

Demographic data (age, sex, medical history) were collected. Ophthalmic evaluations included slit-lamp examination, color Doppler ultrasound, and fundus photography.

### CTA acquisition and CPB assessment

**Figure 1:**
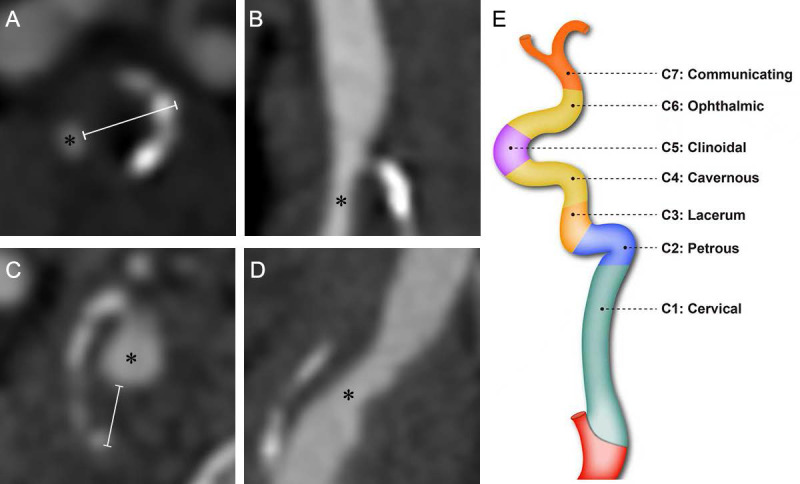
Computed tomography angiography images of the internal carotid artery (ICA) in a patient with diabetes mellitus (DM) (A, B) and a non-DM subject (C, D), demonstrating the method for plaque thickness measurement. Asterisks indicate the residual lumen of the ICA. White lines represent the measurement of maximal plaque thickness. Schematic illustration of internal carotid artery segmentation (E).

Computed tomography angiography (CTA) was performed using a 64-row multidetector CT scanner (LightSpeed VCT; GE Healthcare, Chicago, IL, USA), covering a range from the aortic arch to the skull base. A total of 65 mL of contrast medium (iohexol, 300 mg iodine/mL) was injected via an 18-gauge needle into the antecubital vein using a power injector at a flow rate of 4 mL/s. A smart prep technique was applied to determine the scan start position. The trigger threshold was set at 140 Hounsfield units (HU), and scanning commenced automatically once the contrast enhancement reached this predefined threshold. The scanning parameters were configured as follows: pixel spacing, 0.625 mm; image matrix, 512 × 512; slice thickness, 0.8 mm; table speed per rotation, 39.37 mm/rotation; gantry rotation time, 0.5 s/rotation; and pitch, 0.984 9. Plaque presence was scored with reference to the carotid plaque scoring method proposed by Matangi *et al*. 10 According to the classification system proposed by Bouthillier *et al*. 11, the internal carotid artery (ICA) was divided into seven segments, designated C1 through C7: C1, the cervical segment; C2, the petrous segment; C3, the lacerum segment; C4, the cavernous segment; C5, the clinoid segment; C6, the ophthalmic segment; and C7, the communicating segment. A schematic illustration of the Bouthillier classification is provided in Figure 1E.

Plaque presence in any segment scored one point, yielding a total score (range: 0-4). The rating only requires the presence/absence of plaques, without considering the number of plaques. The plaque thickness score was based on a modification of the carotid plaque thickness grading system proposed by Ihle-Hansen *et al*. 12 The maximum thickness of the plaque within each of the aforementioned ICA segments (C1-C2, C3, C4, and C5) was measured via CTA cross-sectional imaging (Figure 1). Each segment was scored based on the maximum plaque thickness (0, 0-1mm, 1-2mm, 2-3mm, and ≥3mm were given 0, 1, 2, 3 and 4 points, respectively). The scores from all segments of the unilateral internal carotid artery were summated to yield the total maximum plaque thickness score, which ranged from 0 to 16. Additionally, we measured the narrowest diameter of each of the aforementioned ICA segments.

### Fundus photography and retinal vessel measurement

**Figure 2:**
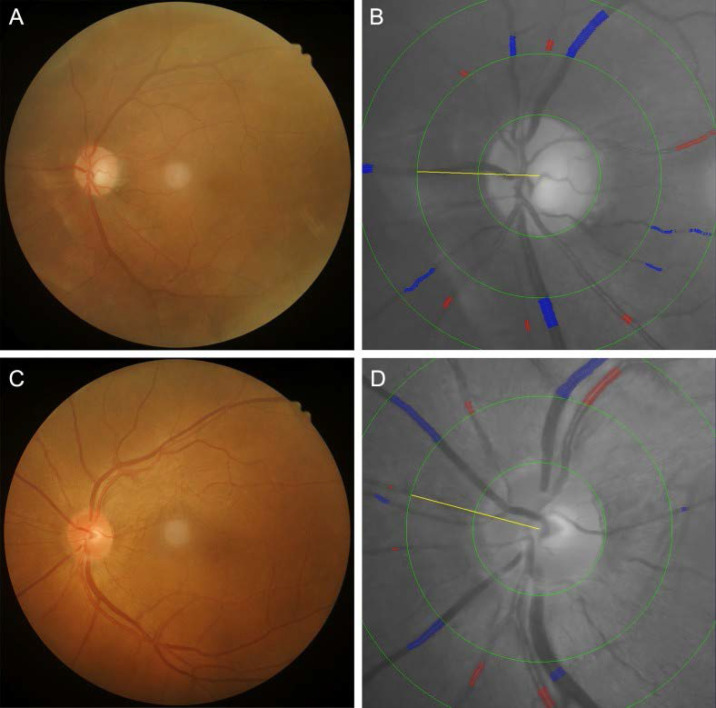
Fundus photographs and corresponding retinal vessel measurements in a patient with diabetes mellitus (DM) (A, B) and a non-DM subject (C, D). In the patient with DM, the central retinal arteriolar equivalent (CRAE), central retinal venular equivalent (CRVE), and arteriolar-to-venular ratio (AVR) were 162.43 μm, 323.21 μm, and 0.503, respectively. In the non-DM subject, CRAE, CRVE, and AVR were 188.92 μm, 337.15 μm, and 0.560, respectively.

Color fundus images centered on the fovea are acquired for all patients using a Kowa fundus camera (Tokyo, Japan) following pupillary dilation and are evaluated by a single trained technician. High-quality images are required to clearly visualize retinal vasculature, the optic disc, and the macula (Figure 2B). Retinal vessel diameters—including the CRAE, CRVE and AVR—are measured semi-automatically using IVAN software (University of Wisconsin, Madison, WI, USA). Fundus photographs are digitized, and the six largest retinal arterioles and venules are analyzed within a zone 0.5-1.0 disc diameters from the optic disc margin. CRAE, CRVE, and AVR are derived using the modified Parr-Hubbard-Knudtson formula, with results reflecting the luminal diameters of the central retinal artery and vein. The reproducibility of these measurements has been previously validated, with intraclass correlation coefficients (ICCs) exceeding 0.85 for all parameters, confirming high interobserver agreement.

### Ocular hemodynamic assessment

Ocular blood flow parameters-including the OA, CRA, and PCA-are evaluated using a color Doppler ultrasound system (MyLab Class C, Esaote, Italy) operated by two certified sonographers. Examinations are performed with a 4-12 MHz linear-array transducer and a fixed sample volume of 1 mm, maintaining an insonation angle of < 20° relative to the vessel axis. During the procedure, subjects are positioned supine with eyes closed. The transducer is gently applied to the upper eyelid without exerting pressure on the globe. Following orbital scanning, optimal Doppler spectra are acquired and stored for analysis. The following hemodynamic parameters are recorded: Peak systolic velocity (PSV), representing arterial filling and perfusion intensity; End-diastolic velocity (EDV), reflecting distal tissue blood supply; Resistance index (RI) and pulsatility index (PI), both quantifying vascular resistance. RI is calculated as (PSV-EDV)/PSV, while PI is derived as (PSV-EDV)/mean velocity. Elevated PI values indicate increased diastolic vascular resistance, whereas higher RI suggests greater peripheral vascular impedance 13. For each vessel, 3-5 consecutive cardiac cycles are analyzed, with total examination time limited to 5 minutes (Figure 2A). Measurement reliability is confirmed by intraclass correlation coefficients >0.85 for all parameters, demonstrating excellent interobserver agreement.

### Statistical analyses

We adopted a two-tiered analytical strategy. For patient-level variables (e.g., age, sex, medical history), comparisons between the diabetes and non-diabetes groups were performed using standard tests for independent samples: the independent samples t-test for normally distributed continuous variables, the Mann-Whitney U test for skewed continuous variables, and the chi-square test for categorical variables. For eye-level variables (e.g., ocular vascular diameters and hemodynamic parameters), we employed linear mixed-effects models (LMM) with a random intercept for each participant to account for the inherent correlation between the two eyes. This approach is the preferred method for handling the nested data structure (eyes nested within patients). In cases where the mixed model failed to converge or encountered a singular matrix fit, we reverted to generalized estimating equations (GEE) with an exchangeable correlation structure and robust standard errors to ensure valid and stable statistical inference. All multivariable models were adjusted for potential confounders, including age, sex, hypertension, smoking history, ischemic heart disease, stroke history, and hyperlipidemia. Statistical significance was set at a two-tailed P-value of < 0.05. All analyses were performed using Python 3.8.

## Results

### Demographic, clinical, and vascular characteristics of participants

A total of 93 eyes from patients with diabetes mellitus (DM; median age 58.0 years; 73.1% male) and 144 eyes from non-DM patients (median age 60.0 years; 69.4% male) were included in this study. The clinical characteristics of the participants are summarized in Table 1. Compared to the non-DM group, the DM group had a significantly higher prevalence of hyperlipidemia (89.2% vs. 66.0%, P < 0.001). No significant differences were observed between the two groups in terms of age, sex, prevalence of carotid artery plaque, hypertension, ischemic heart disease, history of stroke, or smoking history. The minimum diameters of the C1-C2, C3, and C4 segments of the internal carotid artery (ICA) were significantly smaller in the DM group than in the non-DM group. Regarding ocular parameters, the DM group exhibited significantly lower CRAE (145.74±31.03 vs. 155.71±28.93 μm, P = 0.012), lower AVR (0.65±0.12 vs. 0.68±0.13, P = 0.032), lower CRA PSV (8.50 vs. 9.40 cm/s, P<0.001), and higher PCA RI (0.71 vs. 0.68, P = 0.003) compared to the non-DM group. No significant differences were found in other ocular vascular parameters between the groups. Detailed data are presented in Table 1.

### Correlations between internal carotid artery plaque parameters and ocular parameters in DM and non-DM groups

**Figure 3:**
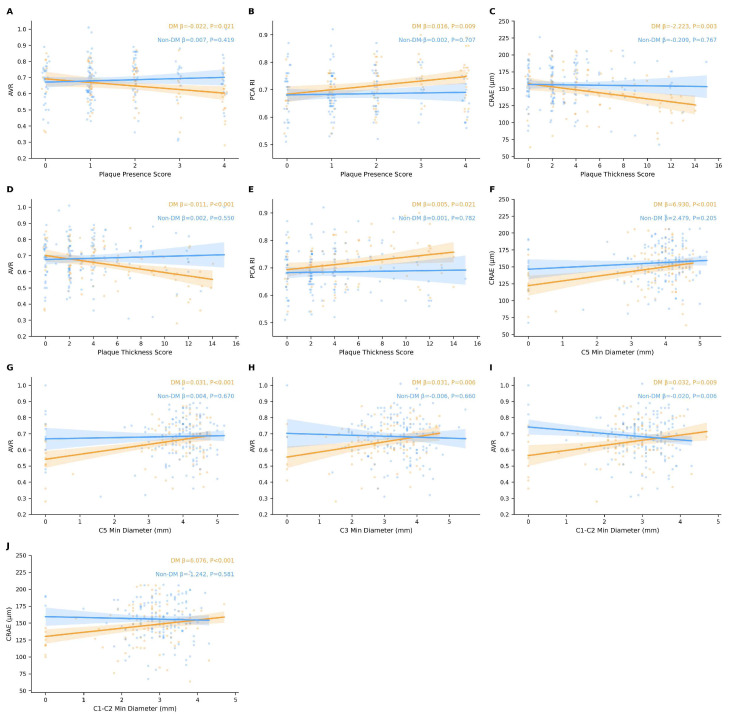
Correlations between ICA plaque parameters (plaque presence score, plaque thickness score, and segmental minimum diameters) and ocular parameters (CRAE, AVR, and PCA RI) in the DM and non-DM groups. Correlation coefficients and P-values were derived from linear mixed-effects models with a patient-level random intercept to account for inter-eye correlation; generalized estimating equations (GEE) with an exchangeable correlation structure were used when mixed models failed to converge. All models were adjusted for age, sex, hypertension, smoking history, ischemic heart disease, stroke history, and hyperlipidemia. *P < 0.05. ICA, internal carotid artery; CRAE, central retinal arteriolar equivalent; AVR, arteriovenous ratio; PCA, posterior ciliary artery; RI, resistance index; DM, diabetes mellitus.

As shown in Figure 3, after accounting for inter-eye correlation using patient-level clustered models and adjusting for extended confounders (age, sex, hypertension, smoking history, ischemic heart disease, stroke history, and hyperlipidemia), distinct patterns of association emerged between carotid plaque parameters and ocular outcomes in the DM versus non-DM groups. In the DM group, the plaque presence score was significantly associated with AVR (beta = -0.022, P = 0.021) and PCA RI (beta = 0.016, P = 0.009). The plaque thickness score was significantly associated with CRAE (beta = -2.223, P = 0.003), AVR (beta = -0.011, P < 0.001), and PCA RI (beta = 0.005, P = 0.021). Segmental analysis of ICA diameters in the DM group revealed that the minimal diameter of the C5 segment was significantly associated with both CRAE (beta = 6.930, P < 0.001) and AVR (beta = 0.031, P < 0.001). The C1-C2 segment minimal diameter showed significant associations with CRAE (beta = 6.076, P < 0.001) and AVR (beta = 0.032, P = 0.009). The C3 segment minimal diameter was significantly associated with AVR (beta = 0.031, P = 0.006). In the non-DM group, no significant associations were observed between plaque thickness score, plaque presence score, or most ICA segmental diameters and the ocular parameters examined (all P > 0.05). However, an unexpected inverse association was detected between the C1-C2 segment minimal diameter and AVR (beta = -0.020, P = 0.006), while the association with CRAE remained non-significant (beta = -1.242, P = 0.581).

### Associations of plaque presence score with ocular parameters: interactions with diabetes status

To further investigate whether diabetic status modifies the associations of plaque thickness score and plaque presence score with ocular parameters, interaction terms were incorporated into linear mixed-effects models accounting for inter-eye correlation, with adjustment for age, sex, hypertension, smoking history, ischemic heart disease, stroke history, and hyperlipidemia (Tables 2 and 3). For PCA RI, DM showed a significant positive association in the main effects model (B = 0.036; 95% CI: 0.013 to 0.058; P = 0.002), while plaque presence score was not significantly associated with PCA RI (B = 0.008; 95% CI: -0.001 to 0.016; P = 0.089). The interaction term (DM × plaque presence score) for PCA RI did not reach statistical significance in the interaction model (B = 0.015; 95% CI: -0.003 to 0.032; P = 0.096). For AVR, although neither plaque presence score nor DM showed significant main effects, the interaction term (DM × plaque presence score) was significant (B = -0.033; 95% CI: -0.060 to -0.007; P = 0.013) (Table 2).

### Associations of plaque thickness with ocular parameters: interactions with diabetes status

Similar trends were observed when plaque presence score was used as the independent variable (Table 3). In the analysis using plaque thickness score (Table 3), the main effects model for CRAE showed that both plaque thickness score (B = -1.288; 95% CI: -2.342 to -0.235; P = 0.017) and DM (B = -8.618; 95% CI: -16.893 to -0.343; P = 0.041) were negatively associated with CRAE. The interaction term (DM × plaque thickness score) remained significant in the interaction model (B = -2.603; 95% CI: -4.678 to -0.528; P = 0.014), indicating that the effect of plaque thickness on CRAE varied by diabetic status. Although the total plaque thickness score was not significantly associated with AVR in the main effects model (B = -0.004; 95% CI: -0.009 to 0.001; P = 0.083), the interaction term (DM × plaque thickness score) was significant in the interaction model (B = -0.014; 95% CI: -0.023 to -0.005; P = 0.002).

## Discussion

The present study identified significant negative associations between carotid plaque burden (CPB) and both CRAE and AVR among individuals with diabetes, whereas no such associations were observed in non-diabetic subjects after adjusting for inter-eye correlation and extended confounders. These findings suggest that diabetic status modifies the relationship between carotid atherosclerosis and retinal microvascular parameters. Our results are partially consistent with previous studies, though some discrepancies warrant discussion. For example, a prospective nested case-control study involving 156 participants reported that AVR and CRAE—but not central retinal venous equivalent (CRVE)—were significantly associated with the risk of carotid plaque formation, independent of body mass index, hypertension, and diabetes 14. This discrepancy may be attributed to two main factors: differences in the comprehensiveness of carotid plaque assessment methods and the specific influence of diabetes on microvascular pathology. First, our study utilized more comprehensive analytical indicators. By incorporating both plaque presence scores and maximum plaque thickness scores, we achieved more sensitive detection of plaque severity and a more accurate representation of the extent of carotid atherosclerosis. This approach is particularly relevant in diabetic populations, where plaque burden tends to be greater and its association with retinal microvascular damage is more pronounced. In contrast, other studies may have relied on relatively simplistic metrics—such as binary ultrasound-based plaque presence—which are insufficient to differentiate variations in plaque burden and severity 15-16. The comprehensive evaluation employed in our study thus enhanced the detectability of the relationship between CPB and microvascular alterations. Second, our findings indicate that diabetes may amplify the association between carotid atherosclerosis and ocular microvascular parameters through distinct pathophysiological pathways, positioning diabetes as an exacerbating factor in vascular disease 17. Hemodynamic disturbances originating from carotid plaques are transmitted via branches of the ophthalmic artery to the retinal and choroidal circulations. Retinal vessels possess autoregulatory mechanisms: in response to decreased ocular perfusion pressure, retinal arterioles initially dilate to maintain blood flow and perfusion. However, beyond a certain threshold, compensatory vasoconstriction occurs 18-19. We hypothesize that diabetic individuals may exhibit impaired vascular autoregulation, potentially rendering them more vulnerable to upstream hemodynamic fluctuations and leading to earlier exhaustion of compensatory reserves. This hypothesis is supported by recent basic research demonstrating that hyperglycemia induces endothelial oxidative stress via upregulation of NADPH oxidase and mitochondrial fission proteins, leading to reduced nitric oxide (NO) bioavailability and impaired endothelium-dependent vasodilation 20. In the retinal circulation, where arteriolar diameter is highly dependent on NO-mediated tone 21, this mechanism may directly contribute to the observed association between CPB and CRAE in diabetic patients.

Regarding the posterior ciliary artery circulation, we observed that diabetic patients exhibited significantly higher PCA RI compared to non-diabetic individuals, suggesting that diabetes itself is associated with increased choroidal vascular resistance. Furthermore, after extended confounder adjustment, plaque thickness score—but not plaque presence score—was significantly associated with PCA RI within the diabetic group, indicating that carotid hemodynamic alterations may preferentially affect choroidal perfusion in diabetic patients. However, in the plaque presence score analysis, the interaction effect on PCA RI was attenuated and no longer reached statistical significance, whereas the interaction effect on AVR remained robust. This discrepancy may reflect that plaque thickness is a more sensitive indicator of hemodynamic compromise than plaque presence, or that the choroidal circulation is influenced by a broader set of factors beyond those captured in our models, consistent with the limited autoregulatory capacity of the choroidal vasculature 22. A potential mechanism that may contribute to these findings involves hemodynamic coupling: carotid stenosis could reduce blood supply through the OA, and diabetic individuals, owing to possibly impaired choroidal autoregulation, may experience more pronounced reductions in EDV and elevations in RI 23. However, given the cross-sectional design, this mechanistic interpretation remains hypothetical and requires confirmation in future longitudinal studies. Previous studies have also indicated that diabetes-related carotid stenosis increases the risk of hypoperfusion and ocular ischemic syndrome 24. Additionally, recent basic research has shown that in type 1 diabetes, endothelial PFKFB3 is further upregulated, resulting in excessive glycolysis that increases lactate production on one hand, and on the other hand exacerbates oxidative stress through Nox1-dependent pathways and activates methylglyoxal-HIF-1α signaling, ultimately associated with impaired endothelium-dependent relaxation. In diabetic patients, the metabolic reprogramming (enhanced glycolysis) induced by disturbed flow and hyperglycemia may have a synergistic effect 25. The choroidal circulation, with its limited autoregulatory capacity, may be particularly susceptible to such metabolic stress 25.

This study also identified a significant negative correlation between the minimum diameter of each carotid segment and both CRAE and AVR in the diabetic group—a relationship absents in non-diabetic individuals. This further supports the hypothesis that diabetes exacerbates vascular pathology, thereby modulating the interaction between carotid stenosis and ocular microcirculation. Experimental studies have demonstrated that high fructose intake induces aortic oxidative stress in rats, characterized by upregulation of NADPH oxidase subunit p47phox and eNOS uncoupling (reflected by a decreased eNOS dimer/monomer ratio), which is associated with reduced NO bioavailability and impaired endothelium-dependent vasodilation 21. These mechanisms may collectively explain the negative correlation between carotid stenosis and CRAE observed in diabetic patients. Prior research has demonstrated that retinal hemodynamic abnormalities in diabetic retinopathy are closely linked to systemic vascular conditions such as carotid atherosclerosis 26. Our results reinforce this concept and suggest that diabetes is associated with more pronounced adverse effects of carotid stenosis on ocular microvascular health. In non-diabetic individuals, neither CPB nor stenosis severity was correlated with the ocular parameters examined. This may imply that non-diabetic subjects retain relatively robust vascular regulatory mechanisms, capable of maintaining ocular perfusion through compensatory vasodilation or collateral circulation 27. Moreover, carotid plaques in non-diabetic individuals may be less frequently associated with microcirculatory impairment, thereby exerting limited influence on ocular hemodynamics. A population-based cross-sectional study also reported stronger correlations between carotid intima-media thickness and retinal microvascular parameters in diabetic patients, with causal implications for structural, functional, and hemodynamic ocular changes in this population 28—consistent with our findings.

In our cohort, diabetic patients exhibited significantly smaller CRAE and AVR values compared to non-diabetic individuals, indicative of early microvascular structural remodeling in diabetes. Hyperglycemia promotes the accumulation of advanced glycation end products, oxidative stress, and inflammatory activation, leading to basement membrane thickening, endothelial dysfunction, arteriolar narrowing, and dysregulated vascular tone 29. The underlying mechanism may involve endothelial-to-mesenchymal transition (EndMT). EndMT is a cellular differentiation process driven by signals such as TGF-β and is involved in the pathogenesis of various chronic vascular diseases 30. Hyperglycemia can elevate TGF-β levels, and TGF-β-induced EndMT is sustained and continuously amplified through a metabolic-epigenetic positive feedback loop. This EndMT process, driven by metabolic reprogramming, is a key mechanism of small artery structural remodeling (e.g., vessel wall thickening) and may explain the more pronounced reduction in AVR observed in the diabetic group 31.

Notably, a previous study by Robyn *et al*. demonstrated that HbA1c was significantly associated with retinal venular diameter but showed minimal association with arteriolar diameter 5, suggesting that glycemic control may have differential effects on arterial versus venous sides of the retinal circulation. This is consistent with our focus on arteriolar parameters (CRAE and AVR) and supports the biological plausibility of our findings despite the absence of HbA1c data. In contrast to our results, one cross-sectional case-control study reported no significant differences in CRAE, CRVE, or AVR between newly diagnosed type 2 diabetes patients and healthy controls 32. Another study described increased CRAE in mild non-proliferative diabetic retinopathy (NPDR) and decreased CRAE in severe NPDR and proliferative diabetic retinopathy (PDR), while finding no significant difference between patients with and without retinopathy 1. These discrepancies may reflect variations in diabetes duration and severity, which are known modifiers of CRAE and AVR 1,32. Our findings further underscore the impact of systemic metabolic dysregulation on ocular microvascular architecture, suggesting that microvascular diameter changes may precede overt diabetic retinopathy. We also observed hemodynamic impairments in the diabetic group. Reduced PSV in the central retinal artery implies diminished retinal perfusion. In diabetic patients, lower PSV may be attributable to increased microvascular resistance 33. Hyperglycemia attenuates endothelial nitric oxide synthesis and promotes calcium overload in vascular smooth muscle cells, resulting impaired vasodilation and increased arterial stiffness 34. Elevated PCA RI reflects heightened distal vascular resistance, likely associated with choroidal microcirculatory dysfunction. Diabetic choroidopathy often involves ischemic changes due to pericyte loss, capillary occlusion, and vascular leakage 35. Increased RI may indicate reduced choroidal flow reserve, supporting the hypothesis that choroidal hemodynamics are compromised early in diabetes. This aligns with previous work; for instance, Divya *et al*. reported significant alterations in resistivity indices and flow velocities in retrobulbar vessels among diabetic patients compared to controls 36, suggesting that PCA RI could serve as a potential biomarker for choroidal ischemia and early diabetic retinopathy. However, the inconsistency in our PCA RI interaction findings across different plaque metrics underscores the need for further research to clarify the relationship between carotid atherosclerosis and choroidal hemodynamics in diabetes.

A key strength of this study is the application of both plaque presence and maximum thickness scores to quantify CPB, combined with rigorous statistical methods (linear mixed-effects models with patient-level random intercepts) to account for inter-eye correlation and extensive confounder adjustment. Our results support clinically relevant distinctions in the management of carotid plaque between diabetic and non-diabetic individuals. Diabetic patients may benefit from intensified monitoring of ocular vascular parameters even when CPB is modest, whereas non-diabetic individuals may be managed based on systemic risk profiles. However, several limitations should be acknowledged. First, the cross-sectional design precludes causal inference; the observed associations cannot establish directionality or temporality. Second, the relatively small sample size, particularly for interaction analyses, may partly explain why the interaction effect on PCA RI in the plaque presence analysis lost significance after extensive confounder adjustment. Third, although we were able to retrieve fasting blood glucose data for the majority of participants, HbA1c, BMI, and detailed diabetes duration data were not available for all participants in this retrospective cohort. Future prospective studies should incorporate comprehensive metabolic profiling to better isolate the independent effects of carotid plaque burden on ocular hemodynamics.

## Conclusion

Diabetes significantly modifies the associations between carotid plaque burden and retinal microvascular parameters (CRAE and AVR), with the modifying effect being more consistent for the retinal circulation compared to the choroidal circulation. These findings underscore the importance of metabolic status in ocular vascular pathology and suggest that diabetic patients may benefit from intensified ocular monitoring even with modest carotid plaque burden. Future studies with larger sample sizes and longitudinal designs are warranted to elucidate the underlying molecular mechanisms and to develop integrated vascular protection strategies tailored to the diabetic population.

## Figures and Tables

**Table 1:** Demographic, clinical, and Vascular Characteristics of Participants

Characteristics	DM (n = 93)	Non-DM (n = 144)	P*
Demographic and clinical characteristics			
Age, y	58.0 (53.5, 66.0)	60.0 (56.0, 69.0)	0.304
Male sex, n (%)	68 (73.1)	100 (69.4)	0.640
Fasting blood glucose (mmol/L)	6.93 (6.09,8.13)	5.29 (4.88,5.69)	< 0.001*
Carotid plaque, n (%)	83 (89.2)	111 (77.1)	0.089
Hypertension, n (%)	70 (75.3)	97 (67.4)	0.738
Hyperlipidemia, n (%)	83 (89.2)	95 (66.0)	<0.001*
Ischemic heart disease, n (%)	34 (36.6)	36 (25.0)	0.090
Stroke history, n (%)	64 (68.8)	91 (63.2)	0.960
Smoking history, n (%)	46 (49.5)	62 (43.1)	0.756
ICA diameters			
C1-C2 min diameter, mm	2.80 (2.30, 3.15)	3.10 (2.60, 3.50)	< 0.001*
C3 min diameter, mm	3.00 (2.30, 3.70)	3.50 (2.83, 3.90)	< 0.001*
C4 min diameter, mm	3.20 (2.70, 3.78)	3.60 (3.00, 4.20)	0.001*
C5 min diameter, mm	3.90 (3.20, 4.30)	4.00 (3.53, 4.30)	0.042*
Ocular vascular diameters			
OA diameter, mm	1.30 (1.20, 1.40)	1.25 (1.20, 1.40)	0.524
CRAE, μm	145.74±31.03	155.71±28.93	0.012*
CRVE, μm	244.75 (207.35, 247.48)	230.90 (214.01, 245.98)	0.518
AVR	0.65±0.12	0.68±0.13	0.032*
Ocular arterial hemodynamic parameters			
OA PSV, cm/s	32.40 (22.90, 42.65)	31.10 (21.45, 38.65)	0.164
OA EDV, cm/s	7.25 (4.66, 10.95)	6.83 (4.70, 9.48)	0.458
OA RI	0.76 (0.70, 0.82)	0.74 (0.69, 0.80)	0.706
OA PI	1.26 (1.10, 1.47)	1.24 (1.10, 1.40)	0.303
CRA PSV, cm/s	8.50 (6.79, 9.83)	9.40 (8.06, 11.48)	< 0.001*
CRA EDV, cm/s	2.30 (1.80, 2.95)	2.50 (1.91, 3.10)	0.107
CRA RI	0.71 (0.67, 0.77)	0.74 (0.67, 0.78)	0.407
CRA PI	1.14±0.18	1.18±0.20	0.111
PCA PSV, cm/s	11.60 (9.80, 14.50)	12.15 (9.51, 15.60)	0.935
PCA EDV, cm/s	3.20 (2.50, 4.20)	3.50 (2.66, 4.59)	0.748
PCA RI	0.71 (0.66, 0.77)	0.68 (0.63, 0.75)	0.003*
PCA PI	1.11 (0.99, 1.24)	1.08 (0.95, 1.21)	0.138

Data were presented as mean ± standard deviation, number (%), or median (25th percentile, 75th percentile). For patient-level variables, comparisons between groups were performed using independent samples t-test, Mann-Whitney U test, or chi-square test as appropriate. For eye-level variables, P-values were derived from linear mixed-effects models with a patient-level random intercept to account for inter-eye correlation. DM, diabetes mellitus; ICA, internal carotid artery; OA, ophthalmic artery; CRAE, central retinal artery equivalent; CRVE, central retinal vein equivalent; AVR, arteriovenous ratio; CRA, central retinal artery; PCA, posterior ciliary artery; PSV, peak systolic velocity; EDV, end-diastolic velocity; RI, resistance index; PI, pulsatility index. *P < 0.05

**Table 2:** Linear mixed-effects / clustered models for associations of plaque presence score, DM, and their interaction with PCA RI and AVR (extended confounder adjustment).

	PCA RI (Main effects) B (95% CI)	PCA RI (Main effects) P	PCA RI (Interaction model) B (95% CI)	PCA RI (Interaction model) P	AVR (Main effects) B (95% CI)	AVR (Main effects) P	AVR (Interaction model) B (95% CI)	AVR (Interaction model) P
Plaque presence score	0.008 (-0.001, 0.016)	0.089	0.001 (-0.011, 0.013)	0.863	-0.005 (-0.019, 0.008)	0.436	0.009 (-0.008, 0.027)	0.301
DM	0.036 (0.013, 0.058)	0.002*	0.008 (-0.032, 0.048)	0.694	-0.032 (-0.068, 0.004)	0.083	0.030 (-0.031, 0.091)	0.331
DM × Plaque presence score			0.015 (-0.003, 0.032)	0.096			-0.033 (-0.060, -0.007)	0.013*
Age	0.002 (0.001, 0.003)	0.003*	0.002 (0.001, 0.003)	0.006*	-0.001 (-0.003, 0.001)	0.2	-0.001 (-0.003, 0.001)	0.295
Sex female1 male0	0.001 (-0.011, 0.012)	0.932	0.001 (-0.011, 0.013)	0.886	0.010 (-0.008, 0.028)	0.295	0.009 (-0.009, 0.027)	0.327
Hypertension	-0.016 (-0.041, 0.008)	0.190	-0.015 (-0.039, 0.010)	0.242	-0.020 (-0.057, 0.017)	0.299	-0.024 (-0.061, 0.013)	0.204
Smoking history	-0.002 (-0.017, 0.014)	0.824	-0.002 (-0.017, 0.014)	0.824	-0.005 (-0.029, 0.019)	0.694	-0.005 (-0.029, 0.018)	0.664
Ischemic heart disease	0.016 (-0.009, 0.040)	0.216	0.014 (-0.010, 0.039)	0.250	0.014 (-0.024, 0.051)	0.478	0.016 (-0.021, 0.054)	0.384
Stroke history	-0.000 (-0.023, 0.022)	0.969	0.001 (-0.021, 0.023)	0.930	-0.011 (-0.044, 0.023)	0.527	-0.014 (-0.047, 0.019)	0.409
Hyperlipidemia	-0.014 (-0.040, 0.012)	0.278	-0.012 (-0.038, 0.014)	0.355	-0.009 (-0.049, 0.031)	0.647	-0.013 (-0.053, 0.026)	0.505

PCA, posterior ciliary artery; RI, resistance index; AVR, arteriovenous ratio; DM, diabetes mellitus. Linear mixed-effects models included a patient-level random intercept to account for inter-eye correlation. *P<0.05.

**Table 3:** Linear mixed-effects / clustered models for associations of plaque thickness score, DM, and their interaction with CRAE and AVR (extended confounder adjustment).

	CRAE (Main effects) B (95% CI)	CRAE (Main effects) P	CRAE (Interaction model) B (95% CI)	CRAE (Interaction model) P	AVR (Main effects) B (95% CI)	AVR (Main effects) P	AVR (Interaction model) B (95% CI)	AVR (Interaction model) P
Plaque thickness score	-1.288 (-2.342, -0.235)	0.017*	-0.031 (-1.472, 1.409)	0.966	-0.004 (-0.009, 0.001)	0.083	0.003 (-0.004, 0.009)	0.407
DM	-8.618 (-16.893, -0.343)	0.041*	2.837 (-9.401, 15.075)	0.650	-0.028 (-0.064, 0.008)	0.125	0.033 (-0.020, 0.085)	0.225
DM × Plaque thickness score			-2.603 (-4.678, -0.528)	0.014*			-0.014 (-0.023, -0.005)	0.002*
Age	-0.269 (-0.728, 0.191)	0.252	-0.234 (-0.688, 0.221)	0.314	-0.001 (-0.003, 0.001)	0.234	-0.001 (-0.003, 0.001)	0.305
Sex female1 male0	2.570 (-1.543, 6.683)	0.221	2.402 (-1.660, 6.464)	0.246	0.010 (-0.008, 0.028)	0.283	0.009 (-0.009, 0.026)	0.320
Hypertension	-5.973 (-14.528, 2.583)	0.171	-6.523 (-14.973, 1.926)	0.130	-0.021 (-0.058, 0.016)	0.263	-0.024 (-0.060, 0.012)	0.195
Smoking history	-0.376 (-5.907, 5.155)	0.894	-0.617 (-6.078, 4.845)	0.825	-0.004 (-0.028, 0.020)	0.735	-0.005 (-0.029, 0.018)	0.652
Ischemic heart disease	4.518 (-4.098, 13.135)	0.304	5.274 (-3.255, 13.803)	0.226	0.014 (-0.023, 0.052)	0.45	0.018 (-0.018, 0.055)	0.326
Stroke history	2.781 (-4.887, 10.449)	0.477	1.922 (-5.686, 9.531)	0.620	-0.010 (-0.043, 0.023)	0.557	-0.015 (-0.047, 0.018)	0.385
Hyperlipidemia	2.813 (-6.344, 11.969)	0.547	2.150 (-6.896, 11.196)	0.641	-0.008 (-0.047, 0.032)	0.708	-0.011 (-0.050, 0.028)	0.577

CARE, central retinal arteriolar equivalent; AVR, arteriovenous ratio; DM, diabetes mellitus. Linear mixed-effects models included a patient-level random intercept to account for inter-eye correlation. *P < 0.05.
